# Individual differences in the early recognition of moral information in lexical processing: An event-related potential study

**DOI:** 10.1038/s41598-017-01623-5

**Published:** 2017-05-03

**Authors:** Qun Yang, Canhuang Luo, Ye Zhang

**Affiliations:** 1 Institutes of Psychological Sciences, Hangzhou Normal University, Hangzhou, 311121 China; 2 Zhejiang Key Laboratory for Research in Assessment of Cognitive Impairments, Hangzhou, 311121 China

## Abstract

Previous studies have shown that intuitive moral cognition occurs at an early stage. However, inconsistent findings indicate that moral information is recognized at a relatively late stage. This study uses the recognition potential (RP) as a neural index and simultaneously measures individuals’ moral preferences using the Moral Foundation Questionnaire. We aim to investigate how individual differences in moral preferences modulate the processing of morality in the pre-semantic stage and provide some insights to explain the variation in rapid information processing linked to morality. The participants performed an implicit task in which recognizable words depicting geographical names or behaviors related to moral, disgusting or neutral content alternated with background stimuli at high rates of presentation. The results showed that the early recognition of moral information manifested in the RP depended on an individual’s moral concerns. Participants with a higher level of endorsement of the harm/care foundation exhibited a greater net moral effect, namely, greater mean amplitudes of the moral-neutral RP difference waves. Meanwhile, only the group that was more sensitive to the harm/care foundation showed a distinctively larger RP for the moral words than for the neutral words. Overall, these findings suggest that the early processing of moral cognition may hinge on individual differences in moral concerns about other people’s suffering.

## Introduction

In traditional moral psychology, moral judgment is featured as a process that involves conscious mental activities and verbal reasoning^1^. Despite rich evidence for the influence of reasoning on moral judgment, a greater number of studies have highlighted the role of intuitive processes^2^.

Having the advantage of high temporal resolution, the event-related potential (ERP) technique has the ability to provide crucial insights into the online processing of moral intuition. Recently, an increasing number of ERP studies have emerged, revealing that different ERP components are associated with early stages of moral cognition. An initial study reported that a larger positivity peaking at approximately 200 ms was observed when participants made rescuing decisions between two relatives than between two strangers endangered by an earthquake^3^. Similarly, a larger P200 was induced by the more unpleasant moral scenarios in another study when the subjects made decisions on two types of moral dilemmas. In addition to the findings that valence ratings on unpleasantness during decision-making are significantly correlated with the P200 amplitudes, the authors noted that this early component may reflect affective processing during the first phase of moral decision-making^4^. A very recent study used more probable moral transgression vignettes and compared the temporal processing of social-moral violations to that of knowledge-based violations. A relatively late positivity was observed starting at 320 ms after the stimulus onset uniquely associated with spontaneous moral evaluation^5^.

In addition, a distinction between prosocial and antisocial behaviors is associated with early components, such as N1 and N2. In particular, behavioral ratings of moral blame have been reported to be negatively correlated with N2 amplitudes^6^. This early moral effect has also been reported in preschool children—early posterior negativity (EPN) was evoked while children watched cartoon characters engaging in moral behaviors, with greater negativities for helping than for harming scenes, and automatic attentional or emotional response was considered to be involved in the early phase of the neural processing of moral evaluation^7^. In another study with adult participants, time-locked neural responses when distinguishing between intentional and accidental harmful actions were found to occur as quickly as 62 ms after the stimulus onset, demonstrating unexpectedly fast responses by the brain to processing morally laden information^8^.

In line with the research noted above, the temporal relation between moral information and purely negative emotion processing has been investigated in one study using lateralized readiness potentials (LRPs) as a neural index in a Go/No-Go paradigm. The results revealed that the participants unconsciously prepared to respond to the moral feature of an act with a “left” or “right” hand selection decision before accessing the physical disgust information that enabled the Go/No Go decision, suggesting that the processing of moral information and physical disgust information may occur at different stages, with an intuitively faster response for moral information^9^. This temporal priority of processing moral information over physical disgust was further verified by another study in which moral conditions, regardless of the degree of disgust emotion involved, were found to evoke greater positivity at ~300–400 ms than non-moral conditions; however, disgust conditions with or without moral content were found to evoke larger positive deflections at ~500–600 ms than non-disgust conditions^10^. The results from these two studies indicate that morally offensive stimuli are processed prior to physically disgusting stimuli, supporting the notion that the early processing of moral cognition may reflect a unique intuition about the right or wrong categorization process instead of pure emotional reactions^10^. However, in a lexical decision task, Luo and colleagues demonstrated that core disgust words are distinguished from neutral words in the early EPN component whereas the features of moral words are accessible in subsequent N320 and N400 components^11^. Similar results have been obtained in two recent ERP studies, demonstrating an early disgust effect when processing pictorial stimuli with moral, disgust or neutral content^12, 13^.

As shown above, there has been growing interest among moral psychologists in clarifying the online processing of moral intuition. However, the evidence to date seems somewhat inconsistent. Various ERP components have been found to be linked to spontaneous moral cognition. Although the time windows for the early stages of moral judgments were approximately ~200–300 ms in some studies^3, 4, 6, 14^, others demonstrated relatively earlier (before 100 ms) or later (after 300 ms) accessibility of automatic moral evaluation in the time-course of processing^5, 8, 10, 11^; even more contradictorily, an early moral effect was detected in some ERP studies prior to a pure emotion effect but was observed after emotional processing in other ERP studies.

In the present study, we attempt to extend the results of previous research by utilizing the recognition potential (RP) as the physiological index to examine how rapidly moral cognition is brought to bear on lexical processing. As indicated by previous research, the RP commonly peaks at approximately ~200–300 ms in the parieto-occipital region in response to meaningful stimuli^11, 15^. The RP is typically obtained by the Rapid Stream Stimulation (RSS) paradigm, which is characterized by the alternate presentation of recognizable and non-recognizable stimuli (background) at a high rate without any inter-stimulus intervals. The rapid rate of stimulus presentation in the procedure (normally, the stimulus onset asynchrony [SOA] of each stimulus is 250 ms) is able to force the participants to process the stimuli with their full attention and to attenuate the contamination of irrelevant variables in the electrophysiological signals of interest^16^. It has been suggested that the RP is linked to the pre-semantic processing of words and that it is particularly related to the processing of semantic categorical features^17, 18^. Several groups of moral researchers have proposed in their work that the early stage of the temporal dynamics in moral judgment may involve a rapid and intuitive “right-or-wrong” categorization process^5, 6, 10^. Thus, we expect the RP component to reflect the categorical moral evaluation in early lexical processing.

In addition, we intend to determine the possible reasons that can account for the variability of the early moral effect. Morality is universal but culturally and individually variable^19^. Evidence suggests that individual differences emerge in the distinct psychological processes involved in moral judgment, such as emotional reaction, controlled cognition and mental-state reasoning^20^. Therefore, a plausible method by which to account for the inconsistency between the existing data may be in terms of a subject’s sensitivity to moral values. Individual variation in moral preferences has been reported to be an important predictor of both behavioral and neural responses in social decision-making tasks^21, 22^.

Moral foundation theory has developed validated measures of personal moral preferences. According to this theory, five universal moral foundations are included: care/harm (relates to an individual’s sensitivity to feel the suffering of others), fairness/cheating (relates to an individual’s sensitivity to issues of unfair treatment, justice and rights), loyalty/betrayal (relates to concerns for group membership and addresses issues such as loyalty, self-sacrifice and betrayal), authority/subversion (relates to issues of maintaining social order and engaging in hierarchical social interactions) and sanctity/degradation (focuses on concerns about physical and spiritual purity, disgust and contamination). Harm and fairness are categorized as the individualizing foundation because they focus on individualizing moral virtues. The other three are identified as the binding foundation because the underlying moral virtues in each of the foundations can bind groups together for greater strength. The Moral Foundation Questionnaire (MFQ) has been developed as a tool to assess a person’s endorsement of the five foundations^23–25^. Cross-cultural studies have tested the five-factor model of moral foundations in independent populations and provided evidence for the universality of the MFQ^26, 27^.

Moral foundations endorsements have been demonstrated to be robustly related to social attitudes and actual behaviors. For example, moral foundation endorsements have been shown to reflect an individual’s political orientation in American culture. In general, political liberals endorse moral values based on the two individualizing foundations, whereas conservatives endorse an additional set of virtues based on the binding foundations^28^. Furthermore, the MFQ is proven to have the ability to predict actual voting behaviors^29^. Additionally, there has been rich evidence indicating that the moral concerns of participants, reflected by MFQ scores, are closely related to individual moral decisions across various contexts. In a recent study, individual differences in multiple moral foundations were found to be predictive of participants’ endorsement of causing harm to one person for the sake of saving more people^30^. In multiple studies designed to examine people’s moral decision-making in video games, the researchers consistently established an association between the participants’ MFQ scores and their moral choices in the fictional game world^31–33^. The relative salience of a given moral foundation is related not only to the reasoning process but also to the likelihood of complying with that foundation in their playing behaviors^31, 32^. However, to the best of our knowledge, few studies to date have investigated the relationship between the relative salience of moral foundations and the neural responses of moral judgments.

To this end, we measured individual participants’ attitudes toward multiple foundational moral values using the MFQ and adopted the RSS procedure to evoke the RP to determine how individual differences in moral preferences are related to the early recognition of moral information in lexical processing. The key prediction is that the degree to which the features of moral information can be attended and recognized at the early semantic processing stage may depend on an individual’s sensitivity to certain moral foundations. Specifically, only for those with a higher level of endorsement of universal moral concerns (such as harm, fairness) may the processing of moral information be of more importance with motivational and affective concerns and thus attract more attention, leading to larger RP amplitudes for moral stimuli compared to physically aversive or emotionally neutral stimuli. Furthermore, the two groups with different moral preferences should exhibit different RP amplitudes induced by moral words.

## Results

**Figure 1:**
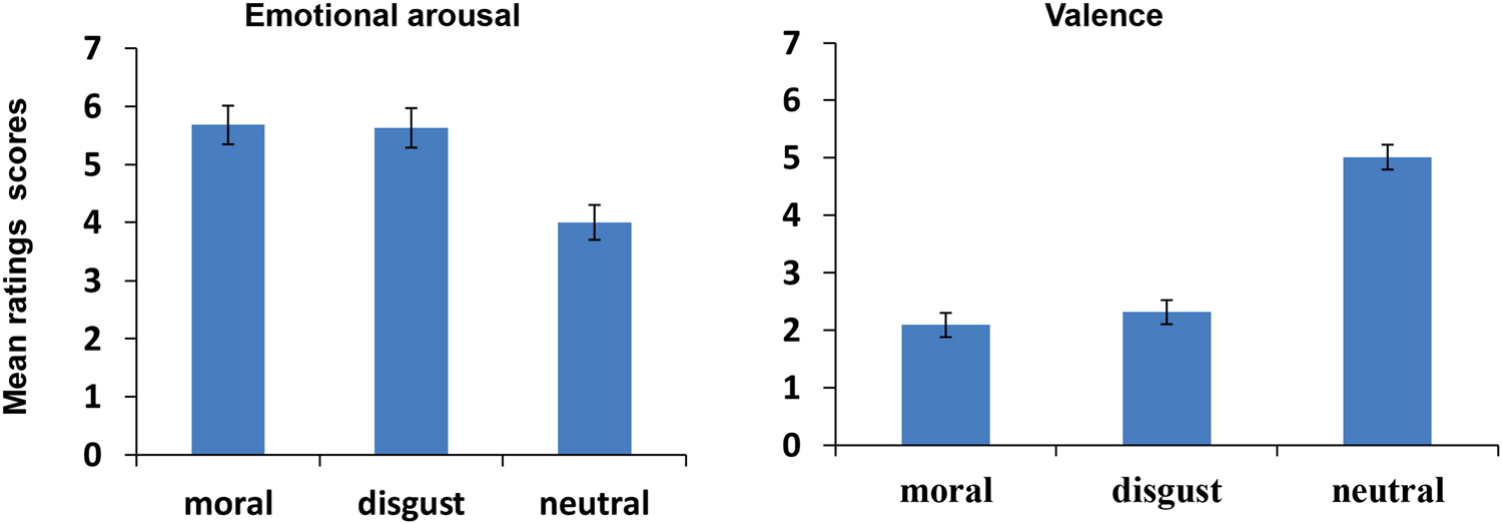
Self-reported rating Scores (M+/−SEM) of the emotional arousal and valence for the moral, disgust and neutral words.

A one-way repeated-measures analysis of variance (ANOVA) with the stimulus types as the within-subject variable was conducted on the rating scores of emotional arousal and valence by the 27 participants from the formal ERP experiment (see Fig. 1). The arousal results showed that there was a significant main effect of the stimulus types (*F*(2, 52) = 10.982, *p* = 0.002, ${\eta }_{p}^{2}$ = 0.297). The moral and disgust words were rated as significantly more emotionally arousing than the neutral words (Bonferroni correction*, p* = 0.004). No differences between the moral words and the disgust words were found. The valence results showed that there was a significant main effect of the stimulus types (*F*(2, 52) = 110.990, *p* = 0.000, ${\eta }_{p}^{2}$ = 0.810). The moral words and the disgust words showed no significant differences in valence, but both were rated as remarkably less pleasant than the neutral words (Bonferroni correction, *p* < *0.001*).

A two-way repeated measures ANOVA with the stimulus types and electrodes as within-subject variables conducted on the mean amplitudes of the RP revealed a significant main effect of the stimulus types(*F*(2, 52) = 4.503, *p* = 0.023, η_*p*_^2^ = 0.148) and a marginally significant main effect of the electrodes and interaction effect between the stimulus types and electrodes (*F*(3, 78) = 3.284, *p* = 0.056, ${\eta }_{p}^{2}$ = 0.112; *F*(6, 156) = 2.146, *p* = 0.066, ${\eta }_{p}^{2}$ = 0.076). The Moral words and the disgust words evoked significantly larger RPs than the neutral words (Bonferroni correction, *p* = 0.07), but did not differentiate from each other.

**Figure 2:**
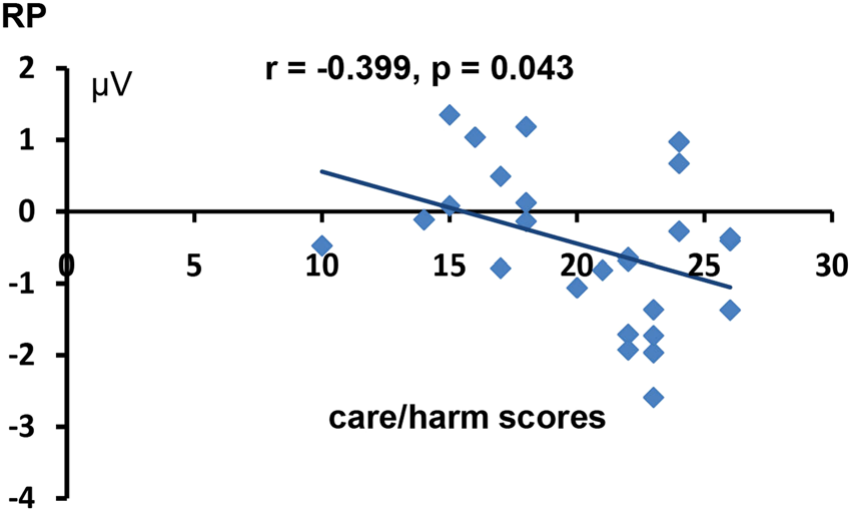
Correlation between the net moral RP effect and the harm/care scores of the MFQ-30.

However, an evident individual variability of the grand-averaged ERP waveforms was observed. Specifically, some participants displayed larger RPs for the moral words relative to the other two types of words, whereas others displayed larger RPs for the disgust words. To further reveal the inter-individual differences of the evoked RPs among all participants, we first computed the net moral or disgust RP effect by subtracting the averaged RP amplitudes of the neutral words across all electrodes from those of the moral words or the disgust words for each participant. Then, we examined the correlations between the net moral or disgust RP effect and their scores on the MFQ-30 and Disgust Scale. The data for one of the participants were excluded from the correlation and the following analysis because the moral RP difference wave amplitudes of this participant went beyond 2.5 standard deviations of the mean number. As shown in Fig. 2, the net moral RP effect was found to be negatively correlated with the Harm/Care foundation scores of the MFQ-30(*r* = −0.399, *p* = 0.043). However, the correlation failed to reach significance when the conservative Bonferroni correction method was applied. We did not observe any other significant correlations.

To further reveal the relation between the participants’ endorsements of moral foundations and the early neural processing of moral information, we used a median split on the Harm/Care scores of the MFQ-30 and assigned the participants who scored above the median score (M = 22) to the high-sensitivity group (N = 14) and the participants who scored below to the low-sensitivity group(N = 12). The scores on each subscale of Moral Foundation Questionnaire and the Disgust Scale for the two groups are presented in Table 1.

**Table 1:** Scores on the Moral Foundation Questionnaire and the Disgust Scale (mean and stand deviation) for the high-sensitivity group and the low-sensitivity group.

	High-sensitivity group	Low-sensitivity group
Total Scores(Disgust Scale)	68.979(10.850)	63.000(12.030)
Pathogen-related	14.929(4.287)	15.0833(4.078)
Body envelope	18.643 (4.781)	16.667(6.652)
Moral	19.357(4.253)	17.083(3.777)
Sex-related	15.857(4.833)	14.167(5.357)
Total Scores(MFQ-30)	95.714(7.956)	83.500(13.028)
Harm/Care	23.571(1.505)	16.583(2.906)
Fairness/Reciprocity	20.857(3.527)	19.250(3.251)
Ingroup/Loyalty	21.571(2.901)	18.917(5.265)
Authority/Respect	18.429(3.204)	17.083 (3.343)
Purity/Sanctity	16.857 (2.797)	16.500(3.371)

**Figure 3:**
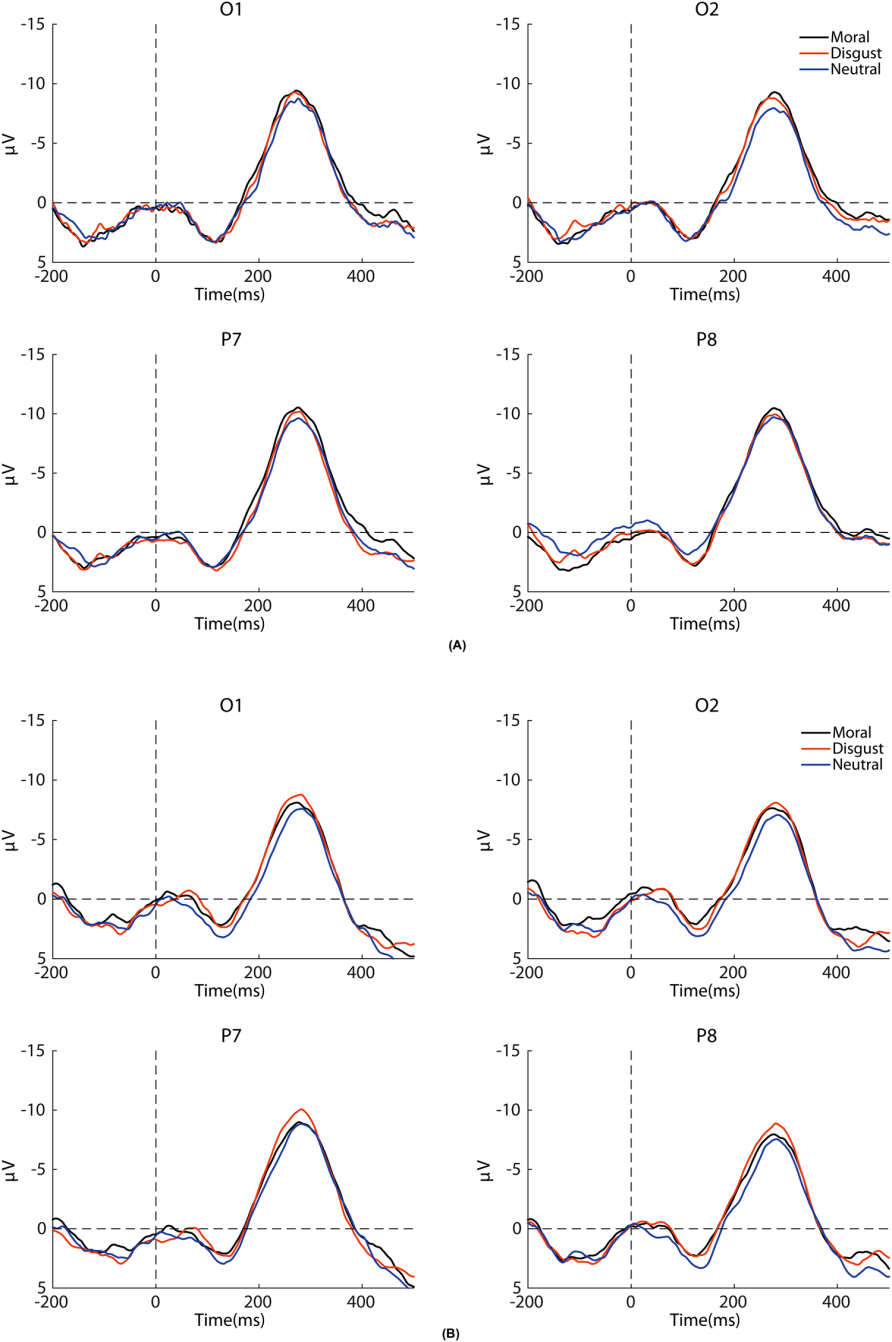
Grand average waveforms for the RP component at O1, O2, P7, and P8 across the three types of words for the high-sensitivity group (**A**) and the low-sensitivity group (**B**).

We then conducted a two-way mixed ANOVA with the stimulus types as a within-subject factor and the group as a between-subject factor on the mean amplitudes of the RP across all electrodes. The main effect of the stimulus types and the interaction between the two independent variables was significant (*F*(2, 48) = 5.667, *p* = 0.011, ${\eta }_{p}^{2}$ = 0.191; *F*(2, 48) = 5.430, *p = *0.013, ${\eta }_{p}^{2}$ = 0.184). The simple effects analysis of the group and the stimulus type interaction was subsequently performed: for the high-sensitivity group, the moral words evoked significantly larger RPs than the neutral words (Bonferroni correction, *p* = 0.002) (see Fig. 3A). No differences between the disgust words and the neutral words were observed; for the low-sensitivity group, the disgust words evoked significantly larger RPs than the neutral words (Bonferroni correction, *p* = 0.016). No differences between the moral words and the neutral words were found (see Fig. 3B).

**Figure 4:**
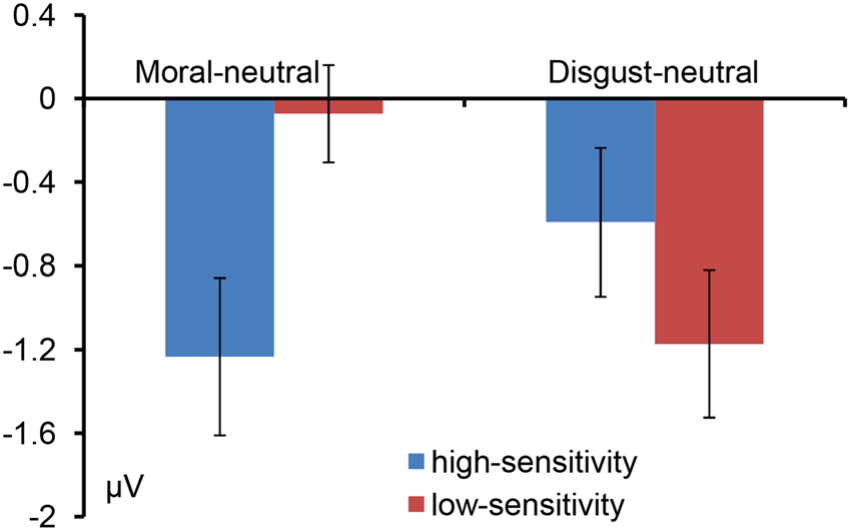
The mean amplitudes of the moral-neutral difference wave for the RP (the net moral effect) and the mean amplitudes of the disgust-neutral difference wave(the net disgust effect) for each group of participants.

Subsequently, we compared the net moral or disgust RP effect between the two groups. The high-sensitivity group demonstrated a significantly larger net moral effect than the low-sensitivity group (t = −2.800, p = 0.01). However, no significant differences for the net disgust effect between the two groups were observed (t = 1.212, p > 0.05) (see Fig. 4).

## Discussion

The present study used the RSS procedure to evoke the RP in an attempt to examine the neural dynamics of moral cognition in the early lexical processing stage, with a special interest in determining whether the early recognition of immorality depends on individual differences in moral preferences. The main results showed that the moral words induced larger RP amplitudes compared to the neutral words. However, this was only true for those participants who were more sensitive to the harm/care moral foundation. The two groups with different levels of endorsement of the harm/care foundation showed distinct early moral effects during the pre-semantic lexical processing.

The RP was initially reported to reflect visual processing based on individual learning experiences^34^. Growing evidence then emerged to support the view that the RP is linked to the pre-semantic processing of images or words. It has consistently been shown that the RP arose in the lower level of word analysis during the reading process whereas its amplitude progressively increased by means of appropriate semantic processing^17, 35^. Moreover, RP amplitudes vary as a function of the semantic categories; for example, the names of animals have been found to display larger RP amplitudes than those of non-animals. As an index of rudimentary semantic categorization, the RP has been verified by adequate evidence^17, 18, 35, 36^. It has been further demonstrated that the RP is strongly influenced by the allocation of attentional resources, with amplified RP in response to attended relative to unattended recognizable stimuli^16^. In addition, emotional states have also been found to be able to modulate the amplitudes of the RP component, with slightly negative emotional states evoking larger RPs than highly negative emotional states^37^.

According to our study, the detection of moral information occurred as early as the pre-semantic processing stage when the participants were only allowed to have a short period of time of 250 ms to recognize each word. As shown from the behavioral results on the emotional arousal and valence, the moral words and the disgust words were rated as equally unpleasant and arousing. However, only the RP amplitudes induced by the moral words were found to be dissociated from those induced by the neutral words in the high-sensitivity group, indicating that the early moral effect cannot simply be attributed to the differences in the emotional states. Instead, we assume that the unique features of immorality spontaneously capture selective attention and hence modulate the early lexical-semantic processing indexed by the RP. More importantly, we find that the early net moral RP effect was related to the harm/care foundation but not to disgust sensitivity, suggesting that the RP induced by the moral words in this study may reflect the spontaneous evaluative processing of the moral status of an act rather than some general affective reaction.

The results from our study add new evidence for the notion that early ERP components are associated with the rapid information processing involved in moral judgment^3, 4, 7–9^. Previous results related to the early stage of moral judgment have been mixed. Similar to our findings, a previous moral study using linguistic materials showed that a word indicating value-based disagreement elicited early neural responses between 200 and 250 ms, revealing the brain’s fast affective responses to morally wrong statements^14^. Other studies using pictorial visual stimuli revealed that early negativities such as N2 and EPN are sensitive to the automatic differentiation between positive and negative moral scenarios^6, 7^. In particular, it has been believed that the EPN effect reflects mechanisms that are similar to the RP component^38^. However, the spontaneous moral evaluation effect has not been reliably detected in early ERP components. Instead, moral information is not accessible until 300 ms after stimulus onset, as demonstrated by some findings^5, 10, 11^. These inconsistencies may be due to several factors, such as the differences in the stimuli or experimental paradigms used in previous research. Among others, the individual differences in moral preferences particularly warrant observation.

As shown by our data, the early detection of immoral words only occurred with some participants. The variation in the early moral effect manifested in the RP can largely be explained by the individual differences in moral preferences in the harm and care foundation. On the one hand, larger RPs were obtained when viewing the moral words compared to the neutral words only for those who had greater moral concerns about the harm/care foundation. On the other hand, those participants who were more sensitive to the pain of others displayed a larger net moral effect than those who were less sensitive. The harm/care foundation is believed to be associated with the ability to feel the pain of others and the virtues of kindness and gentleness^39^. Harm and fairness have been regarded as the core values in moral concerns in traditional moral psychology^24^. Recently, some researchers have gone further to argue for the central role of harm in evaluating immoral behaviors, asserting that perceived harm drives moral judgment^40^. In this study, the sensitivity to the harm and care foundation, we believe, may determine the relative adaptive importance of moral information in the lexical processing of words, which in turn may lead to the varied allocation of “motivated” attention in the early neural responses.

Previously, several ERP studies have documented the relation between the neural correlates of moral evaluation and individual dispositional factors. As indicated in the study by Chiu Loke *et al*. the P3 component, which reflects the participants’ moral reasoning about prosocial-helping behaviors, is related to their self-rating scores on a prosocial personality questionnaire. The more prosocial an individual was, the longer the P3 peak latency that the researchers observed^41^. Similarly, Yoder *et al*. found that the differential amplitudes of late positivities (~300–600 ms) when participants made moral judgments about good and bad behaviors were significantly correlated with individual dispositions in cognitive empathy^6^. However, the ERP-disposition relationship in both of these studies was only observed in late time windows. To the best of our knowledge, the present study is the first to reveal that the individual differences in the harm and care concern are related to the neural processing of moral content in such early ERP components as the RP.

Future research is needed to elucidate the discrepancy in the brain-disposition results between our study and others. One noteworthy factor regarding the interpretations is cultural differences in moral judgment^42^. Given the vast body of research in moral neuroscience, it is surprising that only a few studies have broached the topic of how cultural factors influence the neural processing of moral judgment. A functional magnetic resonance imaging (fMRI) study compared the neural correlates of moral decision-making between Korean and American subjects. Enhanced brain activities in the brain regions were shown to be associated with intuitive judgments for Koreans, whereas they were shown in those brain areas with cognitive control processes for Americans in the moral-personal condition^43^. Another piece of evidence using the ERP method detected neural distinction between personal and impersonal dilemmas in the P3 component between ~280–380 ms for Westerners whereas it occurred in the P260 component between ~200–300 ms for Chinese when all participants needed to make judgments about the appropriateness of an action in hypothetical moral dilemmas^44^. Therefore, it is possible that the present findings may be specific to the population in a certain culture. By all accounts, considering cultural variables that affect the neural processing of moral judgment may be useful to resolve the inconsistencies that have emerged in previous research.

Interestingly, the dissociation between the disgust words and the neutral words was also reflected by the RP in some of the participants. Participants who had lower scores in the harm and care foundation showed significantly larger RP amplitudes for the disgust words than for the neutral words. However, the correlation between the mean RP amplitudes of the disgust-neutral difference wave and the scores on the disgust subscale was not significant. Moreover, we did not find any difference in the net disgust RP effect between the two groups. In fact, we found that the RP amplitudes induced by the disgust words were not much different between the two groups (high-sensitivity group: mean = −9.614; low-sensitivity group: mean = −9.875; no significant differences). Perhaps this result is not only because the participants in the group were slightly more alert to the disgust words but also because they responded with slightly less sensitivity to the neutral words (high-sensitivity group: mean = −9.074; low-sensitivity group: mean = −8.702; no significant differences). At present, however, we can hardly draw any conclusions about the underlying cause of the dissociation between the disgust words and the neutral words.

Nonetheless, what we found in the current research promotes our understanding of the relationship between the processing of moral judgment and that of the emotion of disgust. Behaviors that violate moral rules have been documented to be one of the primary elicitors of disgust^45^. It has been proposed that disgust elicitors have expanded from the physical domain to the socio-moral domain^45, 46^. Not only is moral disgust linguistically analogous to physical disgust, but it may also represent a sub-set of the experience of disgust^47^. In this study, we found that moral and disgust stimuli showed different time-locked neural response patterns in the two groups. Moreover, the moral effect in the RP component was not correlated with behavioral ratings on the disgust sensitivity scale. Along with our previous studies, we consistently support the notion that the evaluation of immoral information is biologically distinct from that of physically disgusting information in the temporal dimension^9, 10^.

### Summary and Limitations

Collectively, we demonstrated that the moral features of lexical words are accessible at the pre-semantic processing stage with individual differences. The early recognition of moral information in the time course hinges on individuals’ moral concerns about harm and care. Only those participants who had a high sensitivity to the suffering of others displayed the ability to distinguish between moral and neutral stimuli in the RP component. Despite these valuable findings, some limitations of this study must be addressed. First, although the moral and disgust stimuli were matched in emotional arousal and valence, the moral words may most likely be considered disgusting by the participants. As argued by previous research, the emotion of disgust can be induced by harmful physical substances and by immoral behaviors^47, 48^. Evidence from previous research and the present study that moral stimuli, regardless of the level of the emotion of disgust, can be temporally dissociable from purely disgusting stimuli relieves some of the concern over this issue^9, 10^. Nonetheless, the failure to separate the disgusting feature from the moral stimuli created a potential confounding factor that may have biased the results. Another concern with the experimental materials is that the moral words that we used were more representative of the harm dimension. Therefore, how the other foundations would be related to the RP if more types of moral words were adopted in the experiment remains inconclusive. Second, despite a strong numerical trend, as expected, the correlation between the harm/care concern and the RP amplitudes of the moral items failed to reach significance when a conservative correction method was applied. Meanwhile, the median split method is normally not a favorable technique for dividing the participants based on some dispositional ratings. In the future, it should be beneficial to use a larger sample to examine the brain-moral dispositions relation. Recruiting more participants, we can screen out two groups with larger moral dispositional differences to replicate the observed results of this study. Finally, due to the characteristics of the experimental paradigm, we were limited to examining the temporal dynamics of the moral judgment in the early components, which made it difficult to compare the findings with those from previous studies since they found the association between brain activities and moral dispositions in later components.

These issues notwithstanding, the results of this research provide new evidence for the fast and intuitive processing of moral information in the early pre-semantic stage. Moreover, the study offers new insights into the causes of the variation in the early moral effect in research.

## Methods

### Participants

Thirty adults were recruited into the study. Three subjects were excluded from further analysis because, for each of them, more than 50% of the experimental trials were removed as a result of artifacts in the data preprocessing (see the Method section for the criteria for artifact removal). Ultimately, twenty-seven adults (17 females) were included in the ERP data analysis. The age of the participants ranged from 18 to 25 years old (M = 21.48, SD = 2.62). All participants were right-handed and had normal or corrected-to-normal vision. No brain damage or neurological or psychological diseases were reported. The experimental procedure of this study was approved by the Institutional Review Board of Hangzhou Normal University, and all methods conformed to the relevant guidelines and regulations with regard to the use of human participants. Each participant signed a written, informed consent form prior to the ERP experiment and was compensated RMB 50 after the experiment.

### Stimuli

The entire set of stimuli consisted of recognizable words and non-recognizable background materials. Four types of words were included as the recognizable materials. Each of the four types of words was composed of three characters. The moral words depicted morally wrong behaviors (e.g., eating a person—); the disgust words depicted morally acceptable but physically disgusting behaviors (e.g., eating rotten meat—); the neutral words depicted both morally and emotionally neutral behaviors (e.g., eating spaghetti—); the target words depicted the name of a city or country (e.g., Hei Long Jiang—). For each set of moral, disgust and neutral words, the first character was the same as a verb, denoting an action, and the last two characters combined as a noun, denoting an object.

The moral, disgust and neutral words used in the formal experiment were carefully selected based on two rounds of pilot studies involving 36 participants (15 males, 21 females). First, the participants were asked to describe whether they think each behavior is morally wrong or whether it is exclusively disgusting. A word was categorized as a moral word if 80% or more of the participants judged a behavior that the word depicted to be morally wrong. A word was categorized as a disgust word if 80% or more of the participants judged the behavior to be exclusively physically disgusting. A word was categorized as a neutral word if 80% or more of the participants judged the behavior to be neither morally wrong nor physically disgusting. Second, the participants were instructed to assess emotional arousal (from very calm to very exciting) and valence (from very unpleasant to very pleasant) using a Likert scale ranging from 1 to 9. Those words having arousal ratings lower than 5 and valence ratings higher than 5 in the moral and disgust categories were excluded. Those words having arousal ratings lower than 5 and valence ratings between 4 and 6 were categorized as neutral words. Moreover, emotional arousal and valence was matched between the moral words and the disgust words. Ultimately, the moral, disgust and neutral categories were composed of 38 words, and another 20 words were included as the target stimuli.

Based on previous RP research, the unrecognizable background stimuli were composed of the recognizable words, which were cut into fragments and restructured using a customized MATLAB script. These background stimuli have no set structures or meanings but were matched in visual attributes with the four types of recognizable stimuli.

Subsequently, 134 recognizable and the corresponding 134 background stimuli constituted the formal set of stimuli for the ERP experiment.

### Procedure

The experiment was conducted in a semi-dark, well shielded and quiet room. The participants were seated at a viewing distance of approximately 60 cm from the computer screen and were instructed to avoid blinking their eyes or moving their bodies as much as possible while keeping their eyes fixated at the center of the computer screen. All stimuli were presented in the center of a 17-inch computer screen using the Song font style with a 28-point font size. The task of this experiment was to respond to the target words. Both the reaction time and accuracy of the behavioral responses were recorded.

**Figure 5:**
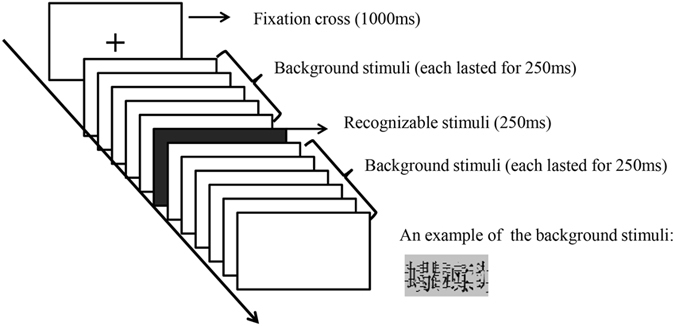
An illustration of the RSS procedure for the stimulus presentation.

At the beginning of a trial, a “fixation cross” was presented for 1000 ms to remind the participants to keep their eyes focused on the center of the screen. Subsequently, 3 to 5 background stimuli were presented. Then, a recognizable word appeared, which was followed by another 4 to 6 background stimuli. Each stimulus lasted for 250 ms (see Fig. 5). The participants were instructed to press the “1” key when they detected the name of a city or a country, namely, the target word. The design of the task is, in fact, irrelevant to the true purpose of the experiment. The implicit task is commonly used in the RSS paradigm^49, 50^. Meaningful target and non-target words were shown to evoke a “fairly equal RP amplitude and same latency”^50^. The recognizable stimuli including target words were presented randomly during the experiment; thus, the participants had to keep their attention on all recognizable stimuli and extract meaning from each recognizable stimulus. Each stimulus of the moral, disgust and neutral words was repeated three times at most. The target words were repeated twice at most. The neighboring background materials within a trial were deliberately made different to prevent visual adaptation.

Prior to the formal experiment, 10 practice trials were arranged to familiarize the participants with the experimental procedure. The participants were only allowed to start the formal experiment when their accuracy rate reached more than 90% in the practice session. The formal test consisted of six blocks of 46 trials each. The participants were allowed to take a short break after each block.

After the ERP experiment, individuals’ moral preferences were measured by the 30-item Moral Foundations Questionnaire (MFQ-30) which is composed of five dimensions: harm/care, fairness/reciprocity, ingroup/loyalty, authority/respect and purity/sanctity. The participants were asked to rate both the moral relevance of 15 foundation-related considerations (e.g., whether someone suffered emotionally—harm/care) and the level of agreement with 15 moral judgment statements (e.g., compassion for those who are suffering is the most crucial virtue—harm/care). The scale has been proven to have fairly acceptable reliability and validity^51, 52^. In addition, to exclude the possibility that the early recognition of moral information could be simply related to a person’s pure emotional sensitivity, the participants were also required to complete the disgust sensitivity scale because disgust is the emotion that is most associated with moral appraisal^53–57^. Disgust sensitivity was assessed using the Chinese Version of the Disgust Scale, which was standardized and validated among Chinese populations by our group on the basis of the Disgust Scale developed by Haidt *et al*.^58^ and the Three Domains Disgust Scale developed by Tybur *et al*.^59^. Four dimensions were included in the Disgust Scale: pathogen-related disgust, body envelope disgust, sex-related disgust and moral disgust. This measure has been shown to have a Cronbach’s α coefficient of 0.92 for the entire scale and values higher than 0.7 for each sub-dimension.

At the end of the experiment, each participant was instructed to rate the arousal and valence for each moral, disgust and neutral word on a scale ranging from 1 to 9.

### Electrophysiological recording and analysis

EEG data were acquired from 32 channels using tin electrodes mounted in an elastic cap (Brain Products GmbH, Munich, Germany). The vertical electrooculogram (VEOG) was recorded with an electrode placed below the left eye, and the horizontal electrooculogram (HEOG) was recorded with an electrode placed next to the orbital rim of the right eye. All inter-electrode impedances were maintained below 5 kΩ. The EEG and EOG signals were amplified using a DC 0.016 ~100 Hz band-pass and were recorded with a sample rate of 1000 Hz. The EEG data were analyzed offline using EEGlab^60^. The data were filtered using a 0.01–40 Hz band-pass infinite impulse response (IIR) filter. Trials with mean EOG voltage exceeding ±75 μV and those contaminated by artifacts as a result of amplifier clipping bursts of electromyographic activity or peak-to-peak deflection exceeding ±75 μV were excluded from further data analysis. ERP averages were computed offline for each type of words; the data were time-locked to the onset of the recognizable words.

Only ERPs elicited by the moral, disgust and neutral words were of interest to us and were thus further analyzed. The mean numbers of remaining trails among all participants for further analysis after data preprocessing were 64 (accounting for 81% of the total trials), 65 (82%) and 63 (80%) for the moral, disgust and neutral conditions, respectively. The averaged epochs for ERPs were 700 ms, which included a 200 ms pre-stimulus and 500 ms post-stimulus activity. Based on the observation of grand-averaged ERP waveforms, a negative component in the parieto-occipital region peaking at approximately 275 ms was elicited by the different types of words. The parieto-occipital distribution is consistent with previous observations^15, 50^. Therefore, the following 4 electrode sites were selected for statistical analysis: O1, O2, P7, and P8. We measured the RP of each individual between 160 and 360 ms after recognizable stimulus onset, in which the RP activity was prominent on average. The p-value was corrected for deviations according to Greenhouse Geisser for all analyses.
